# Repertory to Hæmorrhage of Bowels

**Published:** 1887-12

**Authors:** John V. Allen

**Affiliations:** Phila.


### REPERTORY TO HÆMORRHAGE FROM THE BOWELS.

JOHN V. ALLEN, M. D., PHILA.

HÆMORRHAGE FROM BOWELS.—Ac. ac., Acon., Aga. ph., Aloë, Alco., All. c., Alumn., Alum., Am. carb., Am. caust., Ambra., Ant. cr., Ant. tart., Aphis., Apis., Apor. c., Arn., Arg. n., Ars. h., Art. ab., Arun., Bals., Bart., Bar. c., Bell., Benz. ac., Bol. s., Bry., Calc. c., Calc. p., Canth., Caps., Carb. a., Carb. veg., Carbn. h., Carbn. s., Carg., Casc., Castor., Chil., China, Chlof., Cinnm., Cit. l., Cle., Coff., Coll., Colocy., Colch., Con., Cop., Croc., Crot. p., Cup., Cup. ac., Cup. ar., Cup. s., Cyc., Der., Dor., Dros., Elaps., Euc., Euph., Eupi., Euo., Fer. mur., Fer. jod., Gas., Geni., Graph., Hep., Hydrs., Ind., Iod., Ip., Ir. v., Jug. c., K. bi., K. ba., K. blr., K. chr., K. iod., K. n., Kis., Lau., Led., Lil. tig., Lip., Lob., Lon., Lyc., Manc., Mag. m., Merc., Merc. l., Merc. cyn., Merc. dul., Merc. n., Merc. p. a., Merc. p. v., Merc. sol., Merc. sulcy., Mez., Mur. ac., Nat. carb., Nat. mur., Nat. alfc., Nit. ac., Nx. m., Nux vom., Olnd., Orm., Ox. a., Petrol., Phos., Phyt., Plan., Plat., Pb., Polyp. o., Puls., Pyrth., Rat., Rei., Rhus tox., Ric., Rut., Saba., Sabi., Sac., Sarr., Sec. c., Senec., Sep., Sil., Sin. a., Squ., Stan., Stram.,

Sul., Sul. ac., Tab., Tann., Tarent., Tel., Tep., Tet., Thal., Thuja, Tril., Val., Verat., Vip., Wies., Zn., Zn. s.

HÆMORRHAGE FROM BOWELS.—*With distended abdomen*, Baryta carb.; *bleeding from anus*, Crotulus, Secale, Tereb.; *bleeding from anus*, of pure blood, with urinary symptoms, Canth.; *bleeding from anus*, constant dropping of blood from, Kobalt; *bleeding from anus*, coagulated blood passes from, Stram.; *with anxiety*, Acon., Ars.; *bloody stool, with small white particles, like frog's spawn, Phos.; black stool, with small hard lumps*, Sulph. ac.; *black stool*, Verat. vir., Caps.; clots of blood pass from the anus, Alum.; *dark small clots*, Hamm., Carl.; *in clots*, Castor, Kis., Lip., Merc. cor., Merc. sol., Nat. mur., Nux vom., Phos., Vip., Stram.; *clots at first*, Calc. phos.; *clots pass in masses, painless, with sinking feeling*, Ver. alb.; *clots in, looking like liver*—Vipera acuatia carnita; *cadaverous smelling, involuntary at night*, Rhus tox.; *in children*, Arun.; *dark*, Ars., Crocus, Crotalus; *dark fluid, involuntary, great debility and fainting*, Crot.; *dark, stringy*, Crocus sat.; *decomposed, looking like charred straw*, Lachesis; *daily*, Colocy.; *after exertion*, Millef. and Cinnam.; *with much flatus, seemingly only flatus*, Ruta; *with solid copious fæces*, Ant. c.; *with fæces covered with blood and mucus*, Mag. mur.; *of tenacious mucus, mixed with black blood*, Caps.; *bloody mucus, with frequent painful stool*, Ailanth; *dark bloody mucus, and sore feeling in abdomen*, Arn.; *of dark, bloody mucus, and brown, dry tongue*, Bapt.; *of bloody masses, and anus feels drawn upward*, Plumb.; *during menses*, Amm. mur., Hydrphb.; *after menses*, Graph.; *after checked metrorrhagia*, Acetic acid.; *during micturition*, Merc. sol.; *morning, after getting up, Plantago; offensive*, Ars., Benz. ac., Colch., Ipec., Rhus tox; *painless*, Verat. Alb.; *painless and frequent*, Apis; *painless, blood streaked, like flesh-colored water*, Phos.; *painful, discharge bloody*, Merc. cor.; *red, bright, not clotted, faint from least motion*, Nit. ac.; *streaked with blood*, Erigeron, Colch., Sulph., Thromb.; *spots and streaks of blood*, Podoph.; *slimy, bloody mucus, offensive*, Ipec.; *slimy mucus, with streaks of blood*, Canth., Colocy.; *slimy discharge of bloody matter, with intense pain in rectum*, Doryphora; *spotted with blood*, Nat. carb.; *threads and bloody mucus, very fetid*, Colch.; *in a jerking stream*, Carlsbad; *smell, like fresh blood, that had stood some minutes in a water bath*, Colchic.; *tar-like blood, in large quantities*, Hamm.; *tongue red, smooth, and cracked*, Kali b.; *when walking*, Carb.

HÆMORRHAGE BEFORE STOOL.—Amm. carb., Lobelia; *of black blood*, Merc.

DURING STOOL.—Alum., Amb., Am. carb., Am. mur., Anac., Calc., Carb. veg., Carb. an., Cast., Caust., Coloc., Ferrum, Gambog., Hep., Kali carb., Lach., Lam., Lyc., Merc., Mur. ac., Nat. mur., Nit. ac., Phos., Plat., Puls., Ruta, Selen., Sabina., Sarsap., Sep., Sil., Sulph. ac., Thuja, Zinc., Valer.; *black, thick*, Asar.; *of pure black*, Merc. cyan.; *with constipated stool*, Ledum, Trifolium prat., Psor., Nat. mur.; *in evening*, Calc. carb.; *with large flow*, Amb.; *frequent and profuse*, Anacard; *first part bloody*, Asar.; *gurgling, like water from a bunghole*, Thuja; *with great want of animal heat*, Ledum.; *light colored*, Casc.; *only a little*, Graph., Plantago.

HÆMORRHAGE DURING STOOL.—*With discharge of bloody mucus*, Drosera; *with discharge of blood and mucus, and sensation as if the anus was on fire*, Iris v.; *of pale blood*, Zinc; *pure blood*, Trillium pend.; *with soft stool*, Hep., Lycopod.; with inclination to stool, only blood passes, Abrot.; stool hard (see constipated).

HÆMORRHAGE AFTER STOOL.—Agar., Aloe, Alum., Am. c., Apis, Cala., Casc., Caps., Canth., Calc. phos., Carb. veg., Chelad., Cycl., Grat., Hydras., Kin., Kali b., Kali n., Lach., Lyc., Merc. sol., Mez., Phos., Rhus v., Sel., Sep., Sulph., Sum., Sabina, Scilla, Trif. p.; *with violent burning in rectum and anus*, Tereb.; *with contraction of anus*, Elaps; *of black blood*, Lobelia; *of dark blood*, Viburnum; *hard stool*, Prunus spin.; *with last portion of stool*, Selen.; *red, thin*, Calad.

